# Discordance between Prevalent Vertebral Fracture and Vertebral Strength Estimated by the Finite Element Method Based on Quantitative Computed Tomography in Patients with Type 2 Diabetes Mellitus

**DOI:** 10.1371/journal.pone.0144496

**Published:** 2015-12-07

**Authors:** Nobuaki Kiyohara, Masahiro Yamamoto, Toshitsugu Sugimoto

**Affiliations:** Internal Medicine 1, Shimane University Faculty of Medicine, Izumo, Shimane, Japan; Rensselaer Polytechnic Institute, UNITED STATES

## Abstract

**Background:**

Bone fragility is increased in patients with type 2 diabetes mellitus (T2DM), but a useful method to estimate bone fragility in T2DM patients is lacking because bone mineral density alone is not sufficient to assess the risk of fracture. This study investigated the association between prevalent vertebral fractures (VFs) and the vertebral strength index estimated by the quantitative computed tomography-based nonlinear finite element method (QCT-based nonlinear FEM) using multi-detector computed tomography (MDCT) for clinical practice use.

**Research Design and Methods:**

A cross-sectional observational study was conducted on 54 postmenopausal women and 92 men over 50 years of age, all of whom had T2DM. The vertebral strength index was compared in patients with and without VFs confirmed by spinal radiographs. A standard FEM procedure was performed with the application of known parameters for the bone material properties obtained from nondiabetic subjects.

**Results:**

A total of 20 women (37.0%) and 39 men (42.4%) with VFs were identified. The vertebral strength index was significantly higher in the men than in the women (*P*<0.01). Multiple regression analysis demonstrated that the vertebral strength index was significantly and positively correlated with the spinal bone mineral density (BMD) and inversely associated with age in both genders. There were no significant differences in the parameters, including the vertebral strength index, between patients with and without VFs. Logistic regression analysis adjusted for age, spine BMD, BMI, HbA1c, and duration of T2DM did not indicate a significant relationship between the vertebral strength index and the presence of VFs.

**Conclusion:**

The vertebral strength index calculated by QCT-based nonlinear FEM using material property parameters obtained from nondiabetic subjects, whose risk of fracture is lower than that of T2DM patients, was not significantly associated with bone fragility in patients with T2DM. This discordance may indirectly suggest that patients with T2DM have deteriorated bone material compared with nondiabetic subjects, a potential cause of bone fragility in T2DM patients.

## Introduction

The association between diabetes mellitus and osteoporosis is a great concern for the elderly. Meta-analyses have demonstrated that patients with type 2 diabetes mellitus (T2DM) are at increased risk of hip fracture compared with non-T2DM subjects [1, 2]. The risk of vertebral fractures (VFs) in T2DM patients is significantly elevated despite the elevated bone mineral density (BMD) of T2DM patients compared with nondiabetic subjects [3]. Assessing the bone fragility in patients with T2DM by BMD is difficult because BMD at any site such as the spine, femoral neck and distal radius 1/3 are not significantly associated with the presence of VFs [3] and because the fracture risk of T2DM patients is elevated compared with nondiabetic subjects at any age and BMD T-score [4]. Bone strength is a composite of both BMD and bone quality [5], and these findings suggest that the patients with T2DM might have poor bone quality that is not apparent in the BMD measurements. Thus, tools are needed to assess comprehensively the bone fragility of T2DM patients, especially the bone quality which consists of material properties such as bone collagen as well as structural properties such as bone geometry and microarchitecture.

The finite element method (FEM) is a computational analytical tool for a complex system such as the stress analysis of a structure. *Ex vivo* studies showed that quantitative computed tomography-based FEM (QCT-based FEM) was superior to BMD and QCT alone for assessing the bone strength of vertebrae [6–8]. In addition, *in vivo* QCT-based nonlinear FEM computed by thin-slice imaging produced by multi-detector computed tomography (MDCT) for clinical use had a higher diagnostic power for VFs than BMD in nondiabetic subjects [9, 10]. These advantages suggest that QCT-based FEM may be useful for T2DM patients to determine the bone fragility, which is difficult to find by BMD, [3] because QCT-based FEM can estimate the integrated the bone strength which consists of BMD and bone structure (one of component of bone quality); however, it is unknown whether the bone strength of T2DM patients can be estimated by QCT-based nonlinear FEM.

To clarify this issue, we investigated the relationship between the presence of VFs and the index of bone strength calculated by QCT-based nonlinear FEM in patients with T2DM.

## Subjects and Methods

### Subjects

This cross-sectional study was approved by the ethical review board of our institution the Shimane University Institutional Committee on Ethics (IRB No. 1648) and it complied with the Helsinki Declaration. All of the subjects agreed to participate in the study and provided written informed consent. A total of 146 Japanese patients with T2DM were sequentially enrolled [54 postmenopausal women (age range 47–84 years) and 92 men (age range 51–88 years)]; these patients had been referred to Shimane University Hospital from community clinics for the treatment of diabetes. All of these patients underwent BMD measurements for the diagnosis of osteoporosis and CT scans for screening to exclude malignant neoplasm-induced or functional adrenal tumor-induced diabetes under standard clinical conditions. Subjects who had higher than normal serum creatinine levels (normal range for women, 0.44–0.83 mg/dl; for men, 0.56–1.23 mg/dl) were excluded from the study and were subjects with primary hyperparathyroidism, hyperthyroidism, and rheumatoid arthritis to avoid metabolic bone disorders caused by secondary osteoporosis, or a history of falls or traffic accidents to eliminate the possibility of injury-associated fractures. None of the subjects received drugs or hormones that might affect their bone metabolism, including sex steroids, glucocorticoids, warfarin, bisphosphonates, teriparatide, and denosumab.

### Biochemical measurements

Blood samples from fasting patients were analyzed for hemoglobin A1c (HbA1c) and serum creatinine (Cr) using automated techniques at our hospital’s central laboratory. Serum bone-specific alkaline phosphatase (BAP) and urinary N-telopeptide (uNTX) were measured using commercially available enzyme–linked immunosorbent assays (ELISAs).

### BMD measurements

BMD values for the lumbar spine (L) and the femoral neck (FN) were measured by dual-energy X-ray absorptiometry (DXA) using a QDR-4500 system (Hologic, Waltham, MA). The values were expressed relative to the standard deviation (SD) of age- and sex-matched normal Japanese BMD values provided by the manufacturer (Z-score). The coefficients of variation for the measurements of L-BMD and FN-BMD were less than 1%.

### Ascertainment of fractures

For all of the subjects, conventional thoracic and lumbar radiographs using lateral and anterior-posterior projections were obtained. A VF was diagnosed according to a reduction of **≥** 20% as defined by the Genant visual criteria [11]; observations were made by two investigators who were blinded to each other's readings. If the VF assessments were discordant between two independent investigators, then the film was separately reassessed. If the re-evaluated findings were discordant, then the case was regarded as a non-fracture.

### Computed tomography scanning and QCT-based nonlinear FEM

The FEM procedure in this study was performed according to the protocol of the previous study for nondiabetic subjects [9] in order to compare with the results from this population. QCT data were embedded into an image that was diverted from scans performed by multi-detector computed tomography (the Aquilion 64, Toshiba Medical Systems Corporation, Otawara, Japan) with a calibration phantom equipped with hydroxyapatite rods in the standard condition for clinical practice as follows: a slice thickness of 2 mm, a pixel width of 0.35 mm, a tube voltage of 120 kVp, a tube current of 360 mA, and a 512 x 512 matrix. The FEM procedure was performed as previously described [9]. A cylindrical region of interest (ROI) with a radius of 10 mm and a thickness of 10 mm was placed manually in the vertebral body of the second lumbar spine. Hounsfield unit values in the ROI were converted into equivalent density. A 3-dimensional FEM of the vertebral body without including the spinous process and interspinal disk was constructed with 2-mm tetrahedral elements and 2-mm triangular plates from the CT image using Mechanical Finder software (Mitsubishi Space Software, Tokyo, Japan) [9, 12]. Young’s modulus and the thickness of each triangular plate on the outer surface of the cortical shell were allocated values of 10 GPa and 0.4 mm, respectively. The mechanical properties of each element were calculated from the Hounsfield unit values. The ash density of each voxel was assigned using the linear regression equation created from the values of the calibration phantom. The ash density of each element was determined as the average ash density of the voxels contained in one element. Poisson’s ratio of each element was set to 0.4. Young’s modulus and the yield stress of each element were calculated from the equations proposed for nondiabetic subjects [13] according to a previous study [9].

Complete restraint to all nodes of the lower end of the vertebral model was applied as boundary conditions for the simulation of VF. The bone strength was calculated every 50 N under the condition of uniaxial and uniformly distributed compression to the upper site of the vertebrae. Each element was considered to yield when its Drucker–Prager equivalent stress reached the element yield stress. Failure was defined as the occurrence of the minimum principal strain of the first element with less than -10,000 microstrains. The vertebral yield and VFs were determined by the occurrence of the yield and failure in at least one element. The fracture load was defined as the vertebral strength index.

### Statistical analysis

All of the parameters are presented as the mean ± standard deviation for each group. The statistical analyses were conducted using StatView (Abacus Concepts, Inc., Berkeley, CA, USA). The statistical significance of the continuous variables was determined using the Mann-Whitney *U* test. Multiple regression analyses were performed with significant variables selected from a simple regression analysis to determine the significant independent variables against the vertebral strength index. Multiple logistic regression analysis was performed after adjusting for the variables shown in the tables. *P*-values of less than 0.05 were considered to be significant.

## Results

### Baseline characteristics of the participants

The background data are shown in Table 1. In total, 20 women (37.0%) and 39 men (42.4%) had VFs. Six women (11.1%) and 12 men (13.0%) had grade 2 or 3 VFs and multiple prevalent VFs. There were no significant differences in the age, BMI, duration of T2DM, and serum levels of HbA1c and BAP between the men and women. The urinary levels of N-telopeptide (uNTX) were significantly lower in the men than in the women (*P* < 0.01). Serum creatinine levels, BMD values, and T-scores were significantly higher in the men than in the women (*P* < 0.01). The vertebral strength index was significantly higher in the men than in the women (*P* < 0.01).

**Table 1:** Background data.

	Women	Men
	Mean (SD)	Mean (SD)
No. of subjects	54	92
No. of subjects with VFs	20 [37.0%]	39 [42.4%]
No. of subjects with grade 2 or 3 VFs	6 [11.1%]	12 [13.0%]
No. of subjects with 2 or more VFs	6 [11.1%]	12 [13.0%]
Age (years)	63.4 (8.5)	65.6 (9.8)
BMI (kg/m^2^)	24.9 (5.4)	24.1 (3.8)
HbA1c (%)	9.23 (2.12)	9.14 (2.16)
Duration of T2DM (years)	10.2 (8.7)	11.8 (9.4)
Creatinine (mg/dL)	0.58 (0.13)	0.75 (0.15) **
BAP (U/L)	30.1 (9.5)	27.2 (10.0)
uNTX (nmol BCE/mmol Cr)	57.4 (58.1)	32.0 (15.0) **
L-BMD (g/cm^2^)	0.875 (0.150)	1.071 (0.215) **
T-score	-1.22 (1.35)	0.19 (1.81) **
Z-score	0.47 (1.02)	0.68 (1.24)
FN-BMD (g/cm^2^)	0.635 (0.117)	0.782 (0.144) **
T-score	-1.42 (1.06)	-0.65 (1.14) **
Z-score	0.20 (1.07)	0.50 (1.33)
Vertebral strength index (kN)	4.25 (1.44)	6.02 (2.13) **

**, *P* < 0.01

The data are expressed as the mean ± S.D. The values in parentheses represent the percentages.

VFs, vertebral fractures; BMI, body mass index; T2DM, type 2 diabetes mellitus; BAP, bone-specific alkaline phosphatase; uNTX, urinary levels of N-telopeptide; BCE, Bone Collagen Equivalents; L, lumbar; FN, femoral neck; BMD, bone mineral density

### Correlations of the vertebral strength index with clinical parameters

The factors that affect the vertebral strength index were investigated by a simple regression analysis (Table 2). The vertebral strength index of both genders was significantly and inversely correlated with age and significantly and positively related to the L- and FN-BMD, T score, and Z-scores, except in women. Significant negative associations were observed between the vertebral strength index and creatinine levels in women and uNTX levels in men. Multiple regression analysis that adjusted for age, creatinine, uNTX and spine BMD, which were significantly correlated by simple regression, revealed that age and BMD were independent negative and positive determinant factors for the vertebral strength index in both women (*r* = -0.43, *P* < 0.001; *r* = 0.35, *P* = 0.003) and men (*r* = -0.36, *P* < 0.001; *r* = 0.55, *P* < 0.001) (Table 3).

**Table 2:** Simple regression analysis between the vertebral strength index and the independent variables.

Independent variables	Women		Men	
	*r*	*P*	*r*	*P*
Age (years)	-0.53	< 0.001 **	-0.31	0.003 **
BMI (kg/m^2^)	0.16	0.264	0.15	0.162
HbA1c (%)	0.08	0.563	0.01	0.905
Duration of T2DM (years)	-0.26	0.053	-0.02	0.878
Creatinine (mg/dL)	-0.29	0.030 *	0.12	0.241
BAP (U/L)	-0.19	0.176	-0.15	0.163
uNTX (nmol BCE/mmol Cr)	0.01	0.973	-0.22	0.042 *
L-BMD (g/cm^2^)	0.43	0.001 **	0.54	< 0.001 **
T-score	0.43	0.001 **	0.54	< 0.001 **
Z-score	0.16	0.257	0.5	< 0.001 **
FN-BMD (g/cm^2^)	0.43	0.001 **	0.38	< 0.001 **
T-score	0.45	< 0.001 **	0.38	< 0.001 **
Z-score	0.22	0.112	0.28	0.006 **

*, *P* < 0.01

**, *P* < 0.01

T2DM, type 2 diabetes mellitus; VF, vertebral fracture; BMI, body mass index; BAP, bone-specific alkaline phosphatase; uNTX, urinary levels of N-telopeptide; L, lumbar; FN, femoral neck

**Table 3:** Multiple regression analysis of the clinical parameters that affect the vertebral strength index.

Independent variables	Women		Men	
	*r*	*p*	*r*	*p*
Age (year)	-0.43	< 0.001 **	-0.36	< 0.001 **
Creatinine (mg/dL)	-0.18	0.125	0.06	0.543
uNTX (nmol BCE/mmol Cr)	-0.01	0.995	-0.08	0.398
L-BMD (g/cm^2^)	0.35	0.003 **	0.55	< 0.001 **
	*R* ^*2*^ = 0.44, *P* < 0.01		*R* ^*2*^ = 0.44, *P* < 0.01	

**, *P* < 0.01

L, lumbar; BMD, bone mineral density; uNTX, urinary levels of N-telopeptide; BCE, Bone Collagen Equivalents; *R* ^2^ indicates coefficient of determination

### Assessment of the VF risk between patients with and without VFs

The clinical parameters of the group without VFs and the group with at least one VF did not differ significantly, including the vertebral strength index (Table 4). Significant differences between the group with more severe VFs, such as grade 2 or 3 VFs and multiple VFs, and without VFs were observed only in the female T-score of femoral neck and the male age in the group with grade 2 or 3 VFs, which were significantly lower (*P* < 0.05) and higher (*P* < 0.05), respectively, than those in the group without VFs.

**Table 4:** Comparison of various parameters between the group without vertebral fractures and each group with vertebral fractures by various definitions.

	No VFs	ALL VFs	Grade 2 or 3 VFx	Multiple VFs
	Mean (SD)	Mean (SD)	Mean (SD)	Mean (SD)
**Women**				
No. of subjects	34	20	6	6
Age (years)	63.2 (9.2)	63.6 (7.3)	66 (8.2)	61.7 (6.7)
BMI (kg/m^2^)	25 (5.0)	24.8 (6.2)	22.8 (4.6)	28.6 (7.8)
HbA1c (%)	9.13 (1.95)	9.99 (2.41)	11.12 (3.43)	10.17 (3.29)
Duration of T2DM (years)	10.8 (9.5)	9.3 (7.2)	8.0 (6.3)	12.5 (5.3)
BAP (U/L)	30.9 (10.0)	28.8 (8.8)	26.2 (7.0)	25.0 (4.3)
uNTX (nmol BCE/mmol Cr)	59.4 (71.7)	54.1 (25.4)	65.7 (37.8)	54.2 (36.3)
L-BMD (g/cm^2^)	0.901 (0.143)	0.832 (0.156)	0.791 (0.055)	0.903 (0.235)
T-score	-0.99 (1.29)	-1.61 (1.40)	-1.97 (0.50)	-0.96 (2.10)
Z-score	0.58 (1.01)	0.27 (1.04)	0.25 (0.39)	0.63 (1.55)
FN-BMD (g/cm^2^)	0.656 (0.120)	0.599 (0.106)	0.562 (0.088)	0.674 (0.101)
T-score	-1.21 (1.08)	-1.77 (0.96)	-2.23 (0.62) *	-1.21 (1.02)
Z-score	0.39 (1.08)	-0.11 (1.00)	-0.37 (0.60)	0.35 (0.87)
Vertebral strength index (kN)	4.36 (1.51)	4.06 (1.75)	3.68 (1.02)	4.29 (1.91)
**Men**				
No. of subjects	53	39	12	12
Age (years)	64.3 (10.4)	67.5 (8.7)	71.8 (8.2) *	66.3 (9.8)
BMI (kg/m^2^)	23.9 (4.1)	24.4 (3.4)	24.8 (3.0)	23.7 (3.4)
HbA1c (%)	9.43 (2.27)	8.74 (1.96)	9.23 (2.75)	9.12 (2.43)
Duration of T2DM (years)	11.5 (9.4)	12.3 (9.5)	13.8 (10.5)	12.5 (8.6)
BAP (U/L)	26.3 (10.2)	28.4 (9.8)	30.3 (7.4)	29.4 (8.8)
uNTX (nmol BCE/mmol Cr)	32.9 (15.9)	30.8 (13.8)	31.5 (14.8)	33.7 (16.0)
L-BMD (g/cm^2^)	1.050 (0.212)	1.099 (0.220)	1.038 (0.216)	1.026 (0.162)
T-score	0.01 (1.78)	0.42 (1.85)	-0.11 (1.83)	-0.21 (1.40)
Z-score	0.55 (1.24)	0.86 (1.23)	0.57 (1.13)	0.40 (1.06)
FN-BMD (g/cm^2^)	0.771 (0.148)	0.796 (0.139)	0.716 (0.127)	0.789 (0.104)
T-score	-0.72 (1.17)	-0.54 (1.11)	-1.21 (1.02)	-0.63 (0.86)
Z-score	0.32 (1.38)	0.69 (1.26)	0.16 (1.29)	0.57 (0.99)
Vertebral strength index (kN)	6.13 (2.15)	5.87 (2.12)	5.03 (1.57)	5.60 (1.78)

Mann-Whitney *U* test

* *P* < 0.05, *vs*. No VFs group

The data are expressed as mean ± S.D.

BMD, bone mineral density; VFs, vertebral fractures; BMI, body mass index; T2DM, type 2 diabetes mellitus; BAP, bone-specific alkaline phosphatase; uNTX, urinary levels of N-telopeptide; BCE, Bone Collagen Equivalents; L, lumbar; FN, femoral neck

Next, the association between the presence of VFs and the vertebral strength index was investigated (Table 5). Logistic regression analysis that adjusted for age (Model 1), L-BMD (Model 2), BMI, HbA1c, and duration of T2DM (Model 3) did not reveal a significant relationship between any of the severities of the VFs defined by the grade or number and the vertebral strength index.

**Table 5:** Associations between the presence of vertebral fractures by various definitions and the vertebral strength index.

	Women		Men	
	OR	(95% CI)	OR	(95% CI)
**All Vertebral fractures**				
Crude	0.86	(0.57–1.30)	0.94	(0.77–1.15)
Model 1	0.83	(0.51–1.35)	0.99	(0.80–1.08)
Model 2	0.99	(0.57–1.72)	0.89	(0.68–1.16)
Model 3	1.01	(0.57–1.78)	0.89	(0.70–1.08)
**Grade 2 or 3 vertebral fractures**				
Crude	0.65	(0.30–1.42)	0.75	(0.53–1.06)
Model 1	0.67	(0.28–1.61)	0.84	(0.58–1.20)
Model 2	1.01	(0.36–2.79)	0.79	(0.51–1.23)
Model 3	0.7	(0.20–2.45)	0.75	(0.47–1.21)
**Multiple vertebral fractures**				
Crude	0.97	(0.55–1.73)	0.88	(0.64–1.20)
Model 1	0.87	(0.43–1.77)	0.9	(0.65–1.24)
Model 2	0.86	(0.41–1.84)	0.9	(0.60–1.37)
Model 3	1.04	(0.42–2.56)	0.89	(0.58–1.36)

Odds ratios for vertebral fractures were presented as per 1 kN increase in the vertebral strength index

Model 1: Adjusted for age.

Model 2: Model 1 additionally adjusted for L-BMD.

Model 3: Model 2 additionally adjusted BMI, HbA1c, and duration of T2DM

OR, odds ratio; CI, confidence interval; T2DM, type 2 diabetes mellitus; L, lumbar; BMD, bone mineral density

## Discussion

In contrast to previous reports on the fracture risk in nondiabetic subjects, the vertebral strength index calculated by the QCT-based nonlinear FEM of patients with T2DM was not associated with any prevalent VFs when utilizing material properties that were obtained from a previous study in nondiabetic subjects [13] according to a standard protocol [9, 12].

The estimation of the skeletal strength of the vertebrae by QCT-based nonlinear FEM is well established in non-diabetic subjects. This method could predict failure loads and fracture patterns in cadaver studies [14] and assess the vertebral compressive strength better than BMD or QCT alone in *ex vivo* studies [6–8, 15] because individual geometric and densitometric inhomogeneities such as bone shape, trabecular structure and mineralization obtained by MDCT can be embedded in FEM. The calculation of bone strength by FEM requires Young's modulus and Poisson's ratio, which are components of material properties; however, FEM using a patient’s own material properties has not been achieved because the determination of the material properties requires invasive procedures such as a bending strength test. According to a published protocol [9, 12], the values obtained from nondiabetic subjects [13] were used as the bone material properties of T2DM patients in this study because no specific values are available for patients with T2DM. However, substituting these values may lead to false results. The increased bone content of pentosidine, an advanced glycation end-product, in spontaneous-onset diabetic rats and the elevated serum levels of pentosidine in patients with T2DM have been associated with bone fragility independent of the bone mineral density [16, 17], which suggests that patients with T2DM have poor bone material properties compared with nondiabetic subjects. In addition, a recent study of the direct measurement of bone strength by microindentation revealed that the bone material strength of T2DM individuals was significantly lower than that of the control subjects [18]. Considering these findings, the lack of association between bone strength estimated by QCT-based nonlinear FEM using nondiabetic material properties and actual bone fragility in T2DM patients indirectly suggests that patients with T2DM have deteriorated bone material properties compared with nondiabetic subjects, which may underlie the bone fragility in T2DM.

Insufficient statistical power may be responsible for the failure to observe a relationship between VFs and the vertebral strength index. This study confirmed the significant negative association between age and the vertebral strength index observed in a previous study [9], which indicates that the analytical procedure of QCT-based nonlinear FEM was performed consistently. Based on data from a previous study on the relationship between VFs and the vertebral strength index in nondiabetic participants [9], statistical power analysis was performed using the same values: the difference in the mean values of the vertebral strength index between subjects with and without VFs was 1 kN, and the standard deviation of the vertebral strength index was 0.80. Because patients with diabetes have an increased risk of fracture compared with nondiabetic subjects, the difference in the mean values of the vertebral strength index in the T2DM patients is expected to be the same or greater than that in nondiabetic subjects. Thus, using the values for nondiabetic subjects may lead to an underestimation of the fracture risk in diabetic patients. When the ratios of the number of patients with and without VFs were assumed to be 1 to 8, as in this study, with 80% power at a two-tailed alpha level of 0.05, the required minimum numbers of subjects for one group were 6 to 11, which implies that the present study was statistically appropriate to obtain conclusive results. However, no association was detected between VFs and the vertebral strength index, which suggests that the application of QCT-based nonlinear FEM to evaluate the bone strength of patients with T2DM without modifying the standard protocol presents technical problems.

This study had several limitations. First, it was not population-based nor were all of the participants enrolled from identical populations; thus, a selection bias could have occurred. Furthermore, the sample size was not sufficiently large to enable us to make definitive conclusions. Second, the patients enrolled in this study were treated at Shimane University Hospital, which is a tertiary care center, and thus, they may exhibit more severe cases of T2DM; therefore, our patients might not be representative of the average Japanese patient with T2DM. Finally, this study did not confirm the association between VFs and the vertebral strength index of nondiabetic subjects as well as the difference in the vertebral strength index between participants with and without diabetes because it was important to avoid unnecessary X-ray exposure to nondiabetic subjects.

In conclusion, the present study demonstrated that the presence of VFs in T2DM patients was not significantly associated with the vertebral strength index calculated by QCT-based nonlinear FEM based on an established standard protocol when the parameters derived from nondiabetic subjects were applied to the bone material properties of diabetic patients. This finding suggests that the bone material properties of T2DM individuals could be deteriorated compared to nondiabetic subjects. QCT-based nonlinear FEM can assess the integrated bone strength, which consists of not only bone mineral density but also bone geometry. Therefore, further studies will be necessary to determine the adequate indexes of bone material properties for patients with T2DM and thereby assess the precise bone strength by QCT-based nonlinear FEM.
